# A Technology-Computer-Aided-Design-Based Reliability Prediction Model for DRAM Storage Capacitors

**DOI:** 10.3390/mi10040256

**Published:** 2019-04-17

**Authors:** Woo Young Choi, Gyuhan Yoon, Woo Young Chung, Younghoon Cho, Seongun Shin, Kwang Ho Ahn

**Affiliations:** 1 Department of Electronics Engineering, Sogang University, Seoul 04107, Korea; ghyoon@sogang.ac.kr (G.Y.); sapienthia@daum.net (S.S.); 2 Department of DRAM Sensing & Advanced Analysis, SK Hynix, Icheon 17336, Korea; wooyoung.chung@sk.com (W.Y.C.); younghoon1.cho@sk.com (Y.C.); kwangho.ahn@sk.com (K.H.A.)

**Keywords:** dynamic random-access memory storage capacitor, technology-computer-aided design, reliability, leakage current, time-dependent dielectric breakdown

## Abstract

A full three-dimensional technology-computer-aided-design-based reliability prediction model was proposed for dynamic random-access memory (DRAM) storage capacitors. The model can be used to predict the time-dependent dielectric breakdown as well as leakage current of a state-of-the-art DRAM storage capacitor with a complex three-dimensional structure.

## 1. Introduction

Dynamic random-access memory (DRAM) cells are continuously scaled down to improve the chip density [1]. The cell-size reduction decreases the storage capacitance (*C*_s_), which is crucial for the data retention time and read sensing margin [2]. Therefore, cell-size reduction while maintaining *C*_s_ is one of the most important technical issues in DRAM cell design. Two approaches have been employed to overcome this obstacle: increasing the surface area (*A*) and increasing the dielectric constant (*k*) of the storage capacitor. For the first approach, storage capacitors are converted from planar into three-dimensional (3D) structures to maximize their aspect ratios [3]. Regarding the second approach, various types of high-*k* materials are introduced such as ZrO_2_ [4], TiO_2_ [5], and SrTiO [6], which tends to deteriorate the defect density and bandgap energy [7]. This implies that state-of-the-art DRAM storage capacitors suffer from reliability issues such as leakage current and time-dependent dielectric breakdown (TDDB) [8]. Therefore, it is required to predict the electrical reliabilities of DRAM storage capacitors with complex 3D structures. Several pioneering studies have been carried out to model the leakage currents [9] and TDDB behaviors [10] of high-*k* dielectric films based on a kinetic Monte Carlo (kMC) method; however, the extension to the complex 3D structure is insufficient owing to the very large computational time [11]. In this manuscript, a full 3D technology-computer-aided-design (TCAD)-based reliability prediction model for DRAM storage capacitors is proposed. TCAD provides a faster calculation process than that based on the kMC method and structural changes can also be easily implemented. The proposed model emulates the leakage current and TDDB behavior based on any commercial TCAD simulator as long as it provides 3D structure generation, an electron and hole continuity equation solver, a Poisson equation solver, and physical models including trap-assisted charge transports [12]. Using this model, the percolation simulation can be applied.

## 2. Methodology

**Figure 1:**
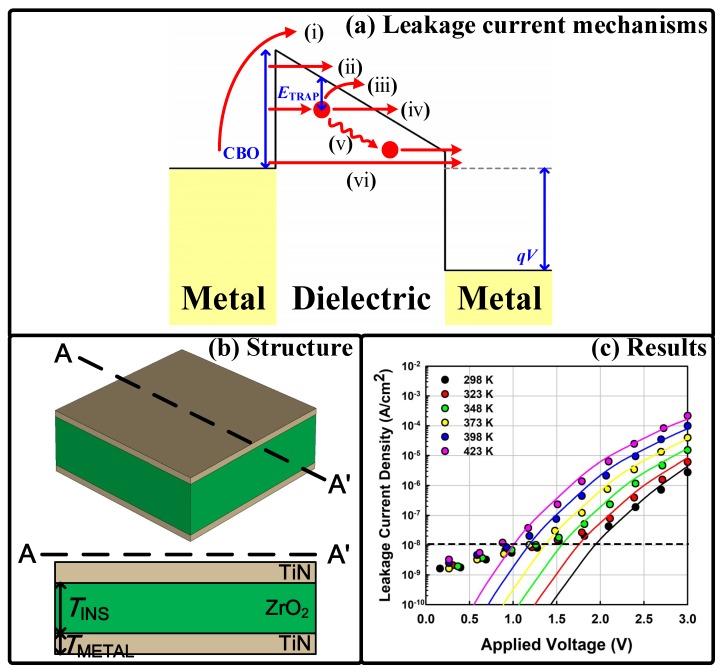
(**a**) Energy band diagram of a metal/insulator/metal (MIM) capacitor, including the possible charge transport mechanisms. CBO: conduction band offset between the metal and dielectric layers. *E*_TRAP_ is an energy level of a trap state. The red arrows show possible charge transport mechanisms: (i) thermionic emission, (ii) Fowler–Nordheim tunneling, (iii) Poole–Frenkel emission (PFE), (iv) trap-assisted tunneling (TAT), (v) trap-to-trap tunneling, and (vi) direct tunneling. The red circles represent trapped electrons at the trap sites. *q* and *V* are the elementary charge and applied voltage, respectively. (**b**) Bird’s-eye and cross-sectional views of a simulated TiN/ZrO_2_/TiN capacitor. (**c**) Calibrated simulation data compared with experimental data [21]. It is worth noting that the leakage current at a low bias is ignored, as only the shallow trap level, which originates from oxygen vacancies, is considered [20].

In this section, the proposed reliability prediction model is explained. The leakage current of a planar DRAM storage capacitor is simulated using the possible leakage current mechanisms of metal/insulator/metal (MIM) structures (Figure 1a): (i) thermionic emission, (ii) Fowler–Nordheim tunneling, (iii) Poole–Frenkel emission (PFE), (iv) trap-assisted tunneling (TAT), (v) trap-to-trap tunneling, and (vi) direct tunneling. Figure 1b shows the structure of the simulated storage capacitor, which has a TiN/ZrO_2_/TiN stack.

The leakage currents of ZrO_2_-based storage capacitors are affected mainly by PFE and TAT owing to the high defect density of ZrO_2_ [13,14]. In addition to the PFE and TAT, the proposed model solved the electron and hole continuity equations coupled with the Poisson equation:

(1) $$\nabla \cdot \left(\varepsilon \nabla \phi \right)=-q\left(p-n+N_{D}-N_{A}\right)-\rho_{\mathrm{trap}}$$

(2) $$\nabla \cdot {\vec{J}}_{n}=q\left(R_{\mathrm{net},n}-G_{\mathrm{net},n}\right)+q\frac{\partial n}{\partial t}$$

(3) $$-\nabla \cdot {\vec{J}}_{p}=q\left(R_{\mathrm{net},p}-G_{\mathrm{net},p}\right)+q\frac{\partial p}{\partial t}$$

where ε is the electrical permittivity, *q* is the elementary electronic charge, *N*_D_ is the concentration of ionized donors, *N*_A_ is the concentration of ionized acceptors, $\rho_{\mathrm{trap}}$ is the charge density contributed by traps, *R*_net,n_ and *R*_net,p_ are the electron and hole net recombination rates, *G*_net,n_ and *G*_net,p_ are the electron and hole net generation rates, ${\vec{J}}_{n}$ is the electron current density, ${\vec{J}}_{p}$ is the hole current density, and *n* and *p* are the electron and hole densities, respectively. The trap-assisted charge transport was calculated using the Shockley–Read–Hall (SRH) recombination rate:

(4) $$R_{\mathrm{net}}=\frac{N_{\mathrm{TRAP}}c_{n}c_{p}\left(np-n_{i}^{2}\right)}{c_{n}\left(n+\frac{n_{i}}{g_{n}}\exp\left(\frac{E_{\mathrm{TRAP}}}{k_{B}T}\right)\right)+c_{p}\left(p+\frac{n_{i}}{g_{p}}\exp\left(\frac{-E_{\mathrm{TRAP}}}{k_{B}T}\right)\right)}$$

where *N*_TRAP_ is the trap density, *E*_TRAP_ is the energy of the trap, *c*_n_ and *c*_p_ are the electron and hole capture rates, and *g*_n_ and *g*_p_ are the electron and hole degeneracy factors, respectively. All of the used tunneling models, such as the elastic/inelastic TAT and trap-to-trap tunneling, are nonlocal models. Only the PFE model was used as a local model and considered to increase the emission rate of electrons injected through tunneling. The electron capture rate for the phonon-assisted (inelastic) transition from the conduction band is [15]

(5) $$\begin{array}{ll} c_{\mathrm{inelastic}}^{n}= & \frac{\sqrt{m_{t}m_{0}^{3}k^{3}T_{n}^{3}}g_{c}}{\hbar^{3}\sqrt{\chi }}V_{\mathrm{TRAP}}S\omega \left\{\frac{\alpha {\left(S-l\right)}^{2}}{S}+1-\alpha \right\}\exp\left\{-S\left(2f_{B}+1\right)+\frac{∆E}{2kT}+\chi \right\}\\ & \;\;\;\;\;\;\;\times {\left(\frac{z}{l+\chi }\right)}^{l}F_{1/2}\left(\frac{E_{F,n}-E_{C}\left(0\right)}{kT_{n}}\right)\frac{{\left|\Psi \left(z_{0}\right)\right|}^{2}}{{\left|\Psi \left(0\right)\right|}^{2}} \end{array}$$

where *V*_TRAP_ is the interaction volume of the trap, *S* is the Huang–Rhys factor, ℏω is the energy of the phonon involved in the transition, α is a dimensionless parameter, *l* is the number of phonons emitted in the transition, *f*_B_ is the Bose–Einstein occupation of the phonon state, $z=2S\sqrt{f_{B}\left(f_{B}+1\right)}$, $\chi =\sqrt{l^{2}+z^{2}}$, ∆E is the dissipated energy, *E*_F,n_ is the Fermi energy, *T*_n_ is the electron temperature, *m*_t_ is the relative tunneling mass, and *g*_c_ is the prefactor for the Richardson constant at the interface or contact. The electron capture rate for the elastic transition from the conduction band is [16]

(6) $$\begin{array}{ll} c_{\mathrm{elastic}}^{n}= & \frac{\sqrt{8m_{t}}m_{0}^{3/2}g_{c}}{\hbar^{4}\pi }V_{\mathrm{TRAP}}{\left[E_{C}\left(z_{0}\right)-E_{\mathrm{TRAP}}\right]}^{2}\Theta [E_{\mathrm{TRAP}}\\ & \;\;\;\;\;\;\;-E_{C}\left(0\right)]\sqrt{E_{\mathrm{TRAP}}-E_{C}\left(0\right)}f\left(\frac{E_{F,n}-E_{\mathrm{TRAP}}}{kT_{n}}\right)\frac{{\left|\Psi \left(z_{0}\right)\right|}^{2}}{{\left|\Psi \left(0\right)\right|}^{2}} \end{array}$$

where f(x)=1/(1+exp(−x)). The electron capture rate for the trap-to-trap tunneling is [17,18]

(7) $$\begin{array}{ll} c_{\mathrm{trap}-\mathrm{to}-\mathrm{trap},i}^{n}= & \underset{j\neq i}{\sum }[C_{f}\frac{\hbar W_{T}\sqrt{\pi }}{m_{t}m_{0}r_{j,i}^{2}Q_{0}\sqrt{kT}}\exp\left(-\frac{W_{\mathrm{OPT}}-W_{T}}{2kT}\right)\exp\left(-\frac{2r_{j,i}\sqrt{2m_{t}m_{0}W_{T}}}{\hbar }\right)\\ & \times \exp\left(-\frac{E_{\mathrm{TRAP}}^{i}-E_{\mathrm{TRAP}}^{j}+\left|E_{\mathrm{TRAP}}^{i}-E_{\mathrm{TRAP}}^{j}\right|}{2kT}\right)f_{j}] \end{array}$$

where transitions occur between a localized state *i* with an energy of $E_{\mathrm{TRAP}}^{i}$ and neighboring localized states *j* with energies of $E_{\mathrm{TRAP}}^{j}$, $W_{\mathrm{OPT}}$ is the trap optical ionization energy, $W_{T}$ is the trap thermal ionization energy, $Q_{0}=\sqrt{2\left(W_{\mathrm{OPT}}-W_{T}\right)}$, *r*_i,j_ is the spatial distance between traps *i* and *j* involved in the transition, *f*_j_ is the localized trap *j* occupation probability, and *C*_f_ is a multiplication factor. The electron capture rate for the PFE model is [17]

(8) $$c_{\mathrm{PFE}}^{n}=\sigma_{\mathrm{PFE}}^{n}v_{\mathrm{th}}^{n}n$$

(9) $$\sigma_{\mathrm{PFE}}^{n}=\sigma_{0}^{n}\left(1+\Gamma_{\mathrm{PFE}}\right)$$

(10) $$\Gamma_{\mathrm{PFE}}=\frac{1}{\alpha^{2}}\left[1+\left(\alpha -1\right)\exp\left(\alpha \right)\right]-\frac{1}{2}$$

(11) $$\alpha =\left(\frac{1}{kT}\sqrt{\frac{q^{3}E}{\pi \varepsilon_{\mathrm{PFE}}}}\right)$$

where $v_{\mathrm{th}}^{n}$ is the electron thermal velocity, $\sigma_{0}^{n}$ is the electron capture cross section, and $\varepsilon_{\mathrm{PFE}}$ is an adjustable parameter. The emission rates were computed following the principle of detailed balance. Figure 1c shows that our simulation results matched well with experimental data under various temperature conditions. The main parameters were: *T*_INS_ = 8 nm, conduction band offset (CBO) = 1.90 eV, *E*_TRAP_ = 1.1 eV, and *N*_TRAP_ = 1 × 10^19^ cm^−3^. It is worth noting that the experimental data measured at a low electric field were ignored in our simulation as they were attributed to deep traps [19]. Only the shallow trap level, which provided the dominant leakage path formed by oxygen vacancies, was considered [20].

**Figure 2:**
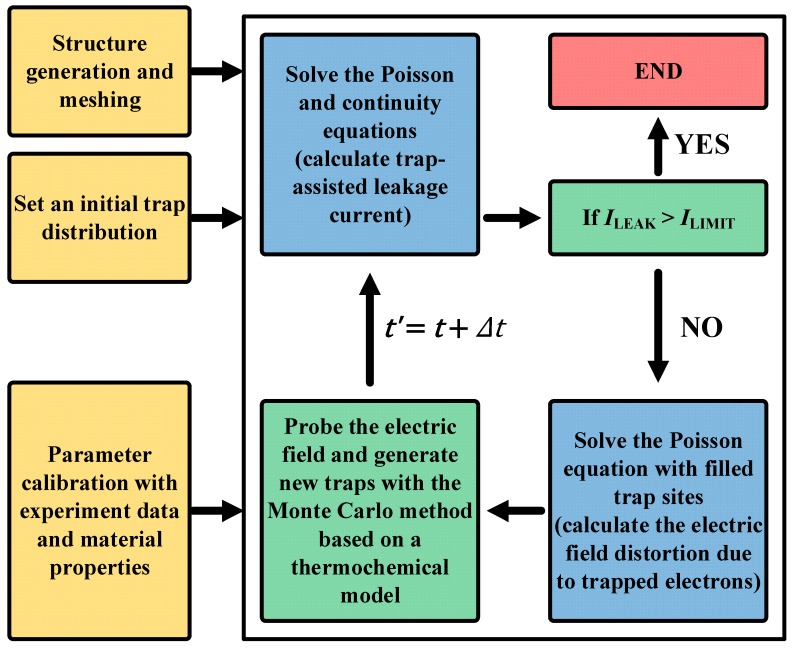
Flowchart of the proposed time-dependent dielectric breakdown (TDDB) model.

The TDDB simulation was performed based on the leakage current simulation. Figure 2 shows a flowchart of the proposed TDDB model. The TDDB simulation followed these five steps: (i) set the initial trap distribution, structure, material parameters, and leakage current at the TDDB condition (*I*_LIMIT_); (ii) after the calculation of the leakage current (*I*_LEAK_) through the trap-assisted charge transport models, determine whether the TDDB condition is satisfied (*I*_LEAK_ > *I*_LIMIT_); (iii) if not, fill the trap sites with electrons and calculate the electric field using the Poisson equation, which is distorted by the trapped electrons; (iv) probe the electric fields of all nodes and calculate the new trap generation probability based on the thermochemical model [8]; and (v) repeat the above procedure after the update of the trap distribution using the Monte Carlo method.

## 3. Results and Discussion

**Figure 3:**
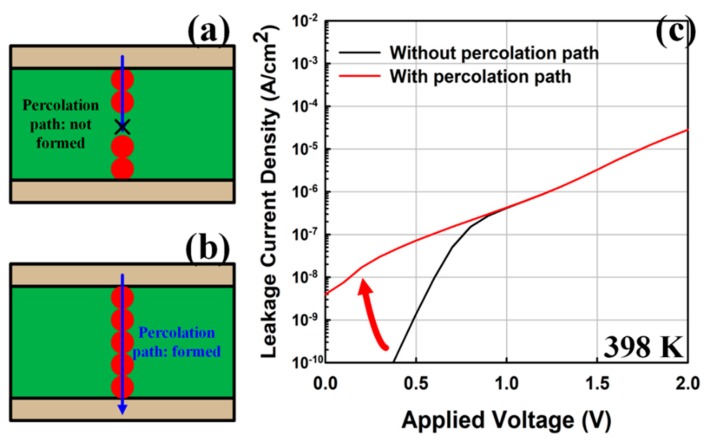
Calculation of *I*_LIMIT_ assuming the percolation path is (**a**) not formed or (**b**) formed; (**c**) corresponding leakage current densities. The red circles represent traps, while the blue lines represent leakage current paths by trap-to-trap tunneling.

**Figure 4:**
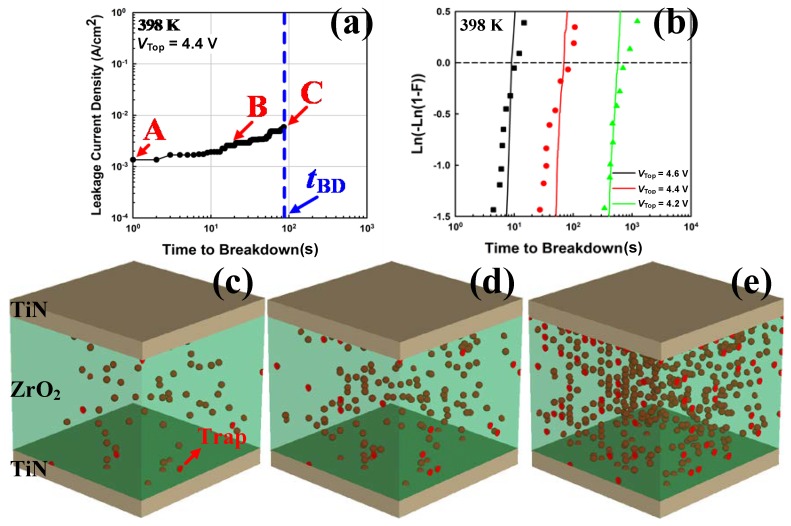
TDDB simulation with a constant voltage stress of 4.4 V at 398 K. (**a**) Leakage current density until the TDDB. (**b**) Weibull plot for the experimental (symbols) and simulation (lines) results. (**c**)–(**e**) represent points A–C in (**a**), respectively. The red spheres indicate trap sites. Trap generation according to the stress and (**e**) formation of a percolation path were observed.

*I*_LIMIT_ was calculated assuming the percolation condition, as shown in Figure 3. In this case, the trap-to-trap tunneling current rapidly increased in the low-bias region, which was used to determine *I*_LIMIT_. Based on the thermochemical model, the probability of bonding breaking (*P*_BD_) is

(12) $$P_{\mathrm{BD}}=\exp\left(-\frac{∆H_{0}}{k_{B}T}+\gamma E\right)$$

where ∆*H*_0_ is the enthalpy of activation for bond breakage, *k*_B_ is the Boltzmann’s constant, *T* is the temperature, *γ* is the field acceleration parameter, and *E* is the applied electric field. The main parameters were ∆*H*_0_ = 1.874 eV and *γ* = 8.67 cm/MV [21]. The newly generated trap was affected by the existing trap. This phenomenon occurred as the existing trap became an electron trap site. The trapped electron reduced the defect formation energy nearby, thus increasing the probability that a new trap would form around the existing trap [22]. The enhancement in the local electric field by the trapped electrons led to an increase in trap generation probability around an existing trap. Figure 4a shows the progression of the trap generation and occupation of TDDB according to the stress. In this case, the stress bias was 4.4 V for the voltage of the top electrode (*V*_TOP_), while the temperature was 398 K. Figure 4c–e show the trap distribution at each point in Figure 4a. Traps were generated through probabilities based on the TDDB model and eventually reached the TDDB condition by forming a percolation path. Furthermore, TDDB simulations were repeated in many samples, changing the stress bias conditions. The TDDB distribution was summarized as a Weibull plot, which was calculated as a cumulative probability density function (CDF), as shown in Figure 4b. Particularly, when ln(–ln(1 − CDF)) = 0, which represents the lifetime, the experimental [21] and simulation results were consistent.

**Figure 5:**
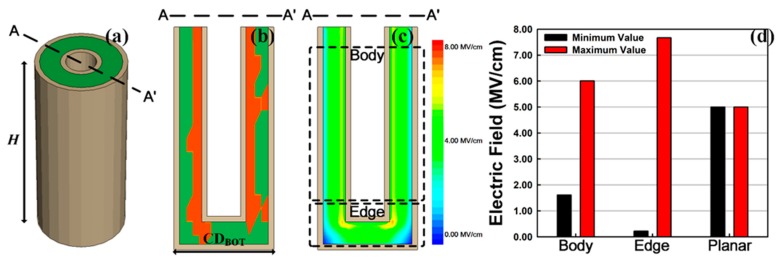
(**a**) Bird’s-eye view of the cylindrical structure. (**b**) Trap formation at the TDDB. The red region indicates generated traps. (**c**) Electric field contour (*V*_TOP_ = 1.0 V, without traps). (**d**) Summarized maximum and minimum electric fields in the body, edge, and planar (*V*_TOP_ = 1.0 V, without traps) cases.

Finally, the TDDB simulation was extended with a 3D cylindrical structure by combining the above simulation results. The structure was defined by: *T*_INS_ = 2 nm, *T*_METAL_ = 0.5 nm, bottom critical dimension (*CD*_BOT_) = 10 nm, and height (*H*) = 20 nm, assuming an extremely scaled and simplified capacitor, as shown in Figure 5a. Figure 5b shows the trap distribution at breakdown. In contrast to the planar structure, the trap formation occurred mainly near the interface between the top electrode and dielectric film. The reason for this is as follows: first, the 3D cylindrical structure shown in Figure 5a had a smaller top electrode than a bottom one, while the planar structure had the same top electrode area as the bottom one. Considering Gauss’s law, this means that the electric field near the top electrode was larger than that near the bottom electrode, which made trap formation near the top electrode easier than near the bottom electrode. Second, the 3D cylindrical structure had two corners in the top electrode while the planar one had no corners. According to the electrostatics, a sharp corner increased the electric field surrounding it. Thus, trap formation was relatively easy around the corners of the top electrode. Figure 5c shows the electric field when *V*_TOP_ = 1.0 V in the absence of a trap, while Figure 5d shows the maximum and minimum electric fields for body, edge, and planar cases. Owing to the nonuniform electric field, considering the cylindrical structure, the maximum electric field was formed at the interface of the top electrode where the Gauss surface was small [23]. This suggests a weakness of top-electrode interface degradation. Particularly, in the edge case, the maximum electric field was enhanced owing to the electric field crowding effect [24]; however, simultaneously, the minimum electric field was significantly reduced owing to the nonuniform electric field. Therefore, in this structure, most samples showed collapse of the body case.

## 4. Conclusions

A full 3D TCAD-based TDDB model for DRAM storage capacitors was proposed. It can be employed to predict leakage current and TDDB in a complex structure, which is required by state-of-the-art DRAM storage capacitors, based on the powerful function of TCAD. In addition, it can be applied to predict characteristic changes due to structural variations, such as surface roughness and etch profile, and can be extended to a mixed-mode and AC analysis by utilizing other functions of TCAD.
